# Relationship between efficiency and clinical effectiveness indicators in an adjusted model of resource consumption: a cross-sectional study

**DOI:** 10.1186/1472-6963-13-421

**Published:** 2013-10-18

**Authors:** Concepción Violán, Oleguer Plana-Ripoll, Quintí Foguet-Boreu, Bonaventura Bolíbar, Alba Aguado, Ruth Navarro-Artieda, Soledad Velasco-Velasco, Antoni Sicras-Mainar

**Affiliations:** 1 Institut Universitari d’investigació en Atenció Primària Jordi Gol (IDIAP Jordi Gol), Av. Gran Via de les Corts Catalanes, 587 Àtic, 08007 Barcelona, Spain; 2 Universitat Autònoma de Barcelona, Barcelona, Spain; 3 Catalan Health Institute, Gran Via de les Corts Catalanes, 587. 08007 Barcelona, Spain; 4 Hospital Dos de Maig, Consorci Sanitari Integral, Dos de Maig, 201. 08025 Barcelona, Spain; 5 Medical Records, Germans Trias i Pujol Hospital, Carretera de Canyet s/n, 08916 Badalona, Spain; 6 Planning Management, Badalona Serveis Assistencials, Via Augusta 9-13.08911 Badalona, Spain

**Keywords:** Adjusted clinical groups (ACG®), Case-mix system, Efficiency, Effectiveness, Risk indicators, Primary health care

## Abstract

**Background:**

Adjusted clinical groups (ACG®) have been widely used to adjust resource distribution; however, the relationship with effectiveness has been questioned. The purpose of the study was to measure the relationship between efficiency assessed by ACG® and a clinical effectiveness indicator in adults attended in Primary Health Care Centres (PHCs).

**Methods:**

Research design: cross-sectional study. Subjects: 196, 593 patients aged >14 years in 13 PHCs in Catalonia (Spain). Measures: Age, sex, PHC, basic care team (BCT), visits, episodes (diagnoses), and total direct costs of PHC care and co-morbidity as measured by ACG® indicators: Efficiency indices for costs, visits, and episodes (*costs EI, visits EI, episodes EI*); a complexity or risk index (*RI*); and effectiveness measured by a general synthetic index (*SI*). The relationship between *EI*, *RI*, and *SI* in each PHC and BCT was measured by multiple correlation coefficients (r).

**Results:**

In total, 56 of the 106 defined ACG® were present in the study population, with five corresponding to 44.5% of the patients, 11 to 68.0% of patients, and 30 present in less than 0.5% of the sample. The *RI* in each PHC ranged from 0.9 to 1.1. Costs, visits, and episodes had similar trends for efficiency in six PHCs. There was moderate correlation between *costs EI* and *visits EI* (r = 0.59). *SI* correlation with *episodes EI* and *costs EI* was moderate (r = 0.48 and r = −0.34, respectively) and was r = −0.14 for *visits EI*. Correlation between *RI* and *SI* was r = 0.29.

**Conclusions:**

The Efficiency and Effectiveness ACG® indicators permit a comparison of primary care processes between PHCs. Acceptable correlation exists between effectiveness and indicators of efficiency in episodes and costs.

## Background

Patient classification systems were introduced more than 20 years ago in order to measure patient characteristics. Of those developed for the hospital environment, Diagnosis Related Groups (DRG) are the most widely reported and used internationally [1]. However, in the primary health care centre (PHC) setting many aspects of these patient classification systems instruments are still in the research phase. Nonetheless, they are beginning to be used in some Spanish regions as an aid to clinical decision-making, health resource planning, resource distribution and epidemiological research, as they allow more reliable and accurate comparisons between physicians than do population characteristics alone [2-4].

Adjusted Clinical Groups (ACG®) [5] is the most widely used of the systems developed for the PHC setting. In this context, age is the variable normally used to adjust resource distribution. However, type of disease may explain half of the variability in the use of resources, measured by frequency (visits or contacts), indirect use (referrals to specialists), and direct costs (diagnostic tests, analyses and drug prescriptions) [6-8].

The Johns Hopkins ACG® System [9] has developed new methodologies to categorize disease types which are closer to the global conception of health status held by PHC physicians and to the determining role that chronic disease may play in resource utilization and clinical management. In addition, predictive models of utilization have been designed that can identify population groups with the potential for very high levels of resource consumption [10,11], classify the types of patients attended, and expedite costs forecasting.

At present, these are the best-validated risk adjustment methods in the Spanish health context. The ACG® can be used for more precise and equitable financial decision-making and to evaluate the efficiency of health resource utilization [12-14]. Analysis of risk-adjustment models in Spain has included the calculation of measures of efficiency. However, no published research to date has assessed whether these indicators are representative of the quality of care provided by PHC professionals (i.e., the effectiveness of the clinical practice). In health care terms, effectiveness is the capacity to achieve a desired or expected effect; efficiency is the capacity to achieve that effect with the minimum viable use of possible resources. Measures of effectiveness in clinical practice derive from a group of indicators defined by evidence-based medicine and/or the various clinical practice guidelines [15-17].

The main objective of this study was to measure the relationship between efficiency (as measured by calculations based on three ACG® values) and effectiveness (a set of process and outcomes indicators) in adult patients attended by PHCs in Catalonia (Spain).

## Methods

### Study design and population

A cross-sectional multi-centre study of computerized medical records of outpatients and several population databases was conducted in 2008 in Catalonia, a region in the northwest of Spain with a population of 7.4 million [18].

The study population consisted of people of both sexes attending 13 PHCs in Catalonia in 2008. Each PHC has at least three (and an average of 12) basic care teams (BCTs), defined as one general practice physician (GP) and one nurse providing care for an assigned set of patients. Each patient is assigned to only one BCT, and the GP is responsible for managing primary care services for that patient, serving as the “gatekeeper” to the national health system. There are four different health services agencies, one of which administers six PHCs and the others four, two, and one centre, respectively. These PHCs have an assigned population of 284,013 inhabitants aged >14 years (56.7% of these aged ≥65 years), served by 187 BCTs. The population is characterized as mainly urban, with a lower-middle socioeconomic level, and predominantly engaged in industry, commerce and services. All four agencies have a modern organization structure, combining public management and private provision of services by agreement with the Catalan Health Service. Policies on staffing, training levels, organization and services offered are representative of most PHC centres in Catalonia, with decentralized management and centralized infrastructure. All of these PHCs use the same electronic health record (EHR) software.

All patients aged >14 years seeking care during 2008 were included, a total study population of 196,593. Patients from other regions or countries, transferring out to other centres or attended only by orthodontists during the study period were excluded.

A ***visit*** was defined as any contact between a PHC team and a patient seeking care due to a health problem, whether in a PHC or at home. An ***episode*** was defined as a process of care for a disease or condition or an explicit patient contact with health services and was coded according to the International Classification of Primary Care (ICPC)-2 [18]. Each episode occurring in the study population was identified by the date it was recorded in the EHR, whether acute or chronic and regardless of when the diagnostic process began. Any one ***visit*** may result in one or more diagnoses of a disease or condition requiring care (***episodes***). On the other hand, one or more ***visits*** may be required to resolve an ***episode*** and to complete the patient’s contact with the BCT resulting from the diagnosis.

Annual coverage (intensity of use) was defined as the ratio of patients attended (196,593) with respect to the assigned population of 284,013 (69.2%). A five-member team (1 information retrieval officer, 2 clinical physicians, 2 consulting technicians) coded the episodes and diagnoses using ICPC-2, then mapped them to an ICD-9-CM for ACG® analysis. The mapping criteria differed according to whether the relationship between the codes was null (one to none), univocal (one to one) or multiple (one to several).

### Patient and medical measures

Patient variables included age, sex, PHC, BCT, number of visits, number of care episodes and total direct costs of PHC care and co-morbidity.

### Model of costs and use of resources

The design of the partial costs system was based on the characteristics of the PHCs. The cost per patient attended during the study period served as the unit of analysis for the final calculation. This methodology is based on the resources used in the visit (referrals, prescriptions, laboratory tests) and indirect costs of a visit (facilities, administration, personnel). The methodology used to calculate the costs was published in the study protocol [14].

### Measures of efficiency and complexity

Adjusted clinical groups (ACG®) were used to obtain three indices of efficiency (*EIs*) and one index of complexity, or risk index (*RI*). The ACG® Grouper (version 8) functional algorithm (http://acg.jhsph.org) is composed of a series of consecutive steps that result in 106 ACG®, which were mutually exclusive for each patient [7,8]. Construction of the ACG® required age, sex and diagnoses coded according to ICD-9-CM [7,8,14].

#### Efficiency indices

Efficiency was evaluated by three indices:

a) *costs EI*: was calculated as the ratio of observed to expected quantity costs.

b) *visits EI*: was calculated as the ratio of observed to expected quantity of visits

c) *episodes EI*: was calculated as the ratio of observed to expected quantity of episodes.

These three indices reflect the relative efficiency of each centre or BCT. Expected episodes, visits and costs were determined indirectly, based on the average number of visits, episodes or costs per patient in an ACG® across all PHCs. The information required for this calculation is the number of visits per ACG® patient in the total study population and the distribution of this reference population in each PHC or BCT to obtain the “expected” numbers as indicated by the average for the 13 PHCs.

The first step was to calculate for each PHC and for each BCT the number of patient visits to be expected in each ACG® if the standardized average number of visits in the reference population for that ACG® is applied. The number of patients in each ACG® category in each PHC and BCT was multiplied by the average number of visits in each category in the reference population. The second step was to calculate the average visits per assigned resident of the catchment area of each PHC and BCT, obtained by dividing the observed number of visits in each case by its assigned population. This same calculation was used to establish the *costs EI* and *episodes EI.*

Any *EI* value equal to one signifies efficiency equal to the 2008 reference population standard, whereas *EI* <1 symbolizes greater efficiency (inverse relation).

#### Risk index (RI)

Defined by the ratio between average expected visits in a PHC or BCT and average number of visits of the reference population [11], an *RI* value equal to one signified a health complexity equal to the 2008 standard, whereas an *RI* >1 represented greater complexity and <1 weaker complexity. The *RI* reflected the complexity of cases attended by a PHC or BCT with respect to the reference population standard. The number of average expected visits for each PHC or BCT was obtained indirectly, based on the average number of visits of the total population in each ACG group.

### Measures of effectiveness

The synthetic index (SI) was obtained from a selection of 20 primary care process and outcomes indicators developed by CatSalut (Catalan Health Service). Originally obtained from the literature, the indicators were subsequently validated by an expert committee. These indicators reflect current standards for procedures related to primary and secondary prevention, diagnosis, treatment and patient monitoring (Table 1). They address selected health objectives defined in the management contract between CatSalut and agencies providing primary healthcare services to the Catalan population [19]. For the implementation criteria, feasibility in the clinical setting was taken into account. The scores for indicators obtained from EHRs range from 1 to 100, reflecting a range from the lowest to the highest effectiveness. For more detail about the indicators and the construction of the SI, see Additional files 1 and 2, respectively.

**Table 1:** **Components of the general synthetic index (SI): definition of each indicator of process and results**[19]

**Indicator**	**Description**
1	Acceptable blood pressure control (<140/90 mm Hg, except in diabetes patients: <130/80 mm Hg.
2	Acceptable diabetes control, with a target of glycated haemoglobin (HbA1c) <7%
3	Blood pressure screening
4	Diabetes mellitus screening
5	Cardiovascular risk calculation in patients aged 35–74 years with total cholesterol >200 mg/dl
6	Alcohol consumption screening (adult population)
7	Ex-smokers, at one year
8	Ischemic heart disease patients with adequate antiplatelet treatment
9	Ischemic heart disease patients with low density lipoprotein <100 mg/ml
10	Cardiac arrhythmia patients with atrial fibrillation and anticoagulation treatment
11	Population >74 years attended and assigned, including home health care
12	Population >74 years attended and assigned, including home health care, with evaluation
13	Flu vaccine administered in the assigned population aged 60 years or more
14	Patients with chronic obstructive pulmonary disease and pneumococcal vaccine administered
15	Use of generic pharmaceutical specialties
16	Use of new medications with limited added value
17	Average cost per defined daily doses of proton pump inhibitors
18	Average cost per defined daily dose of statins
19	Average cost per defined daily dose of angiotensin-converting enzyme inhibitors (ACEI) and angiotensin II receptors antagonists (Angio II)
20	Average cost per defined daily dose of serotonin re-uptake inhibitors and new-generation antidepressants

### Statistical analysis

All data were carefully reviewed before beginning the statistical analysis, with researchers observing frequency distributions and searching for possible errors in recording or coding. Initial descriptive analysis of the results was obtained by classifying patient sociodemographic and clinical characteristics by PHC. Results were reported using the mean and standard deviation or median and interquartile rank for continuous variables and percentages for categorical ones. Descriptive analysis of episodes, total costs and visits for each ACG® reported the mean and standard deviation; the ACG® distribution in each PHC was described using absolute numbers and percentages. The *EI* was calculated for episodes, costs, and visits, each generating an index for comparative analysis; the *RI* and general *SI* were calculated for each BCT and PHC. The relationship between the *EIs*, *RI* and *SI* in each BCT was measured by a multiple correlation coefficient (r). Statistical significance was established as *P =* 0.05. The analysis used SPSS v18, Stata/SE 11.0 for Windows, and R version 2.10.1.

### Ethics and clinical research

The study protocol was approved by the Committee on the Ethics of Clinical Research of the Jordi Gol i Gurina Foundation of the University Institute for Research in Primary Care (*Institut Universitari d’Investigació en Atenció Primària Jordi Gol*, IDIAP)*.* Data were obtained in electronic format and the confidentiality of records was respected at all times according to Spanish law LO 15/1999 13 December, Protection of personal data privacy (*Protección de Datos de Carácter Personal*).

## Results

### Description of population and variables

A total of 196,593 inhabitants were included in the analysis (69.2% coverage). Baseline characteristics of the 13 PHCs are presented in Table 2. The average age was 49.9 (standard deviation [SD] 19.9, range 45.5-53.0) and 56.7% were female (range 51.8%-60.0%). Three of the PHCs had a median of 3.0 episodes per patient and 10 centres had a median of 4.0, with an Interquartile Rank ranging from 2.0 to 7.0. The lowest average cost per patient was 527.2€ and the highest was 807.8€, with a mean of 702.4€.

**Table 2:** Characteristics of patients attending family medicine services

Assigned population, n	284,013
Attended population, n	196,593
Annual coverage (%)	69.2%
PHC Centres, n	13
BCT, n	187
Age in years	49.9 (19.9)
Attended population >65 years, n (%)	52,381 (26.6)
Attended females, n (%)	111,427 (56.7)
Number of episodes/patient	4.5 (3.3) ; 4 [2-6]
Number of visits/patient	7.8 (8.1) ; 5 [3-10]
Number of laboratory tests/patient	2.5 (3.4) ; 1 [0–4]
Number of radiographs/patient	1.2 (1.9) ; 0 [0–2]
Number of complementary tests/patient	0.2 (0.5) ; 0 [0–0]
Number of referrals to specialist/patient	0.7 (1.1) ; 0 [0–1]
**Costs per patient, €**	
Visits	184.7 (191.2)
Laboratory tests	57.7 (76.7)
Radiographs	23.1 (35.5)
Complementary tests	7.0 (20.7)
Referrals to specialist	76.6 (121.0)
Pharmaceutical costs	353.4 (719.3)
*Total Primary Health Care costs*	*702.4 (896.5)*

Data are mean (standard deviation); median [IQR] unless otherwise indicated.

PHC: Primary Health Care Centre.

BCT: Basic care team.

The distribution of ACG®, along with the average number of episodes, costs and visits for each ACG®, is presented in Table 3. A total of 56 of the 106 defined ACG® were present in 196,593 patients, with 5 corresponding to 44.5% of the patients, 11 to 68.0% of patients, and 30 present in less than 0.5% of the population.

**Table 3:** Characteristics of episodes, costs, visits and distribution of patients in each ACG®

		**Population**	**Episodes**	**Total costs**	**Visits**
**ACG® code**	**ACG® description**	**n = 196,593**	**mean (SD)**	**mean (SD)**	**mean (SD)**
4100	2-3 other ADG combinations, age 35+	28,864 (14.7)	3.9 (1.3)	776.3 (828.2)	7.0 (5.2)
300	Acute minor, age 6+	23,095 (11.7)	1.7 (0.9)	173.2 (249.8)	2.9 (2.6)
4910	6-9 other ADG combinations, age 35+, 0–1 major ADGs	14,876 (7.6)	10.2 (2.4)	1,624.4 (1,092.9)	17.4 (10.3)
4410	4-5 other ADG combinations, age 45+, no major ADGs	10,551 (5.4)	6.6 (1.6)	1,025.4 (755.9)	10.8 (6.2)
4420	4-5 other ADG combinations, age 45+, 1 major ADGs	10,137 (5.2)	6.7 (1.6)	1,336.2 (1,061.6)	12.2 (8.7)
2100	Acute minor/likely to recur, age 6+, w/o allergy	9,689 (4.9)	3.4 (1.4)	310.3 (296.3)	5.3 (3.8)
500	Likely to recur, w/o allergies	8,815 (4.5)	1.6 (0.8)	192.6 (274.4)	2.6 (2.3)
400	Acute major	7,511 (3.8)	1.6 (0.8)	243.2 (388.8)	3.0 (2.6)
1800	Acute minor/acute major	6,993 (3.5)	3.4 (1.3)	355.9 (393.2)	5.7 (4.0)
1600	Preventive/administrative	6,937 (3.5)	1.1 (0.3)	259.7 (540.2)	2.0 (2.2)
900	Chronic medical: stable	6,175 (3.1)	2.0 (0.7)	512.8 (580.5)	4.1 (3.3)
3900	2-3 other ADG combinations, males age 18 to 34	5,877 (2.9)	3.4 (1.0)	341.7 (399.1)	5.6 (4.1)
3200	Acute minor/acute major/likely to recur, age 12+, w/o allergy	5,535 (2.8)	5.4 (1.8)	529.0 (477.1)	8.1 (5.2)
2300	Acute minor/chronic medical: stable	5,345 (2.7)	3.7 (1.3)	634.8 (594.8)	6.8 (5.0)
3600	Acute minor/acute major/likely to recur/chronic medical: stable	5,300 (2.7)	7.8 (2.3)	1,043.4 (742.9)	12.4 (7.2)
4310	4-5 other ADG combinations, age 18 to 44, no major ADGs	4,168 (2.1)	5.9 (1.3)	554.8 (474.6)	9.3 (5.1)
4920	6-9 other ADG combinations, age 35+, 2 major ADGs	4,089 (2.0)	10.6 (2.5)	2,102.5 (1,407.8)	20.7 (13.9)
	Other ACG® codes	32,636 (16.6)	5.0 (4.0)	747.8 (1,045.4)	9.0 (10.0)

Data are n (%) unless otherwise indicated.

### Efficiency, risk and effectiveness in primary health centres

Efficiency, risk and effectiveness indices for each PHC are described in Table 4. In six PHCs, all three *EI* results (*costs EI, visits EI, episodes EI*) pointed in the same direction (i.e., higher or lower than 1); the remaining seven centres had differences between these ratios*. Costs EI* ranged from 0.80 to 1.14, *episodes EI* from 0.94 to 1.07 and *visits EI* from 0.89 to 1.23. Therefore, the episodes variable differed the least between expected and observed data. The *RI* ranged from 0.89 to 1.09: the PHC population was 11% less complex than the standard at the lower end of the range, and 9% more complex at the upper end. The *RI* was greater than 1 in seven centres and below 1 in four centres. Finally, the *SI* ranged from 46% to 64%, with most of them between 53% and 56%.

**Table 4:** Efficiency, risk index and effectiveness for each primary health care centre in the study

	**PHC1**	**PHC2**	**PHC3**	**PHC4**	**PHC5**	**PHC6**	**PHC7**	**PHC8**	**PHC9**	**PHC10**	**PHC11**	**PHC12**	**PHC13**
**Efficiency indices**													
Expected costs/patient, €	733.65	752.71	728.61	686.39	707.07	691.07	721.16	757.56	695.46	637.15	711.20	638.55	664.07
Observed costs/patient, €	775.79	600.87	752.10	772.50	807.85	693.66	694.88	757.02	693.06	678.34	666.05	648.02	527.16
*Cost efficiency index*	1.06	0.80	1.03	1.13	1.14	1.00	0.96	1.00	1.00	1.06	0.94	1.01	0.79
Number expected episodes/patient	4.73	4.80	4.93	4.57	4.66	4.59	4.46	4.61	4.56	3.98	4.46	4.23	4.49
Number observed episodes/patient	4.61	4.88	5.09	4.59	4.93	4.72	4.42	4.94	4.31	3.76	4.30	4.14	4.60
*Episodes efficiency index*	0.98	1.02	1.03	1.00	1.06	1.03	0.99	1.07	0.94	0.95	0.96	0.98	1.02
Number expected visits/patient	8.17	8.33	8.49	7.85	8.02	7.90	7.80	8.09	7.87	6.93	7.76	7.29	7.71
Number observed visits/patient	8.93	8.56	9.04	9.05	9.87	9.31	7.17	7.90	7.94	5.40	6.39	6.85	8.72
*Visits efficiency index*	1.09	1.03	1.07	1.15	1.23	1.18	0.92	0.98	1.01	0.89	0.99	0.93	1.13
**Risk index**	1.05	1.07	1.09	1.00	1.03	1.01	1.00	1.03	1.01	0.89	0.99	0.93	0.99
**Effectiveness index**													
*Synthetic index*	50.67	59.19	54.93	55.53	56.62	56.72	46.18	55.56	50.09	53.25	59.42	56.57	64.09

Expected data computed as indirectly standardized rates taking the total population as a standard population.

*PHC:* Primary Health Care Centre.

### Relationship between efficiency, risk and effectiveness for basic activity units

There was a moderate correlation between the *costs EI* and *visits EI* (r = 0.59, P < 0.001) but only a weak correlation between *episodes EI* and *visits EI* (r = 0.17, P = 0.021) or *costs EI* (r = −0.12, P = 0.099). The correlation between the RI and the other indices was significant only for the episodes EI. The correlations between SI and the EIs were significant for episodes and cost (r = 0.48 and r = −0.34, respectively) (Table 5).

**Table 5:** Relationship between efficiency, risk and effectiveness indices for 187 basic care teams*

	**Cost EI**	**Episodes EI**	**Risk index**
**Visits EI**	**0.59** (<0.0001)	**0.17** (0.02)	
**Risk index**	−0.10 (0.16)	**0.54** (<0.0001)	
**Synthetic index**	**−0.34** (<0.0001)	**0.48** (<0.0001)	**0.29** (<0.0001)

*The relationship between indicators was measured by multiple correlation coefficients (r).

In bold, significant values (*P* < 0.05). In parentheses, *P* value.

EI: Efficiency index.

## Discussion

The *episodes EI* and *costs EI* calculated with the ACG® system had an acceptable correlation with an indicator of effectiveness (*SI*). *Visits EI* and *costs EI* also had adequate correlations between them.

The ACG® helps to measure the health status of a population from the perspective of the burden of morbidity and resource utilization. This study analysed various indicators used to assess resource utilization (i.e., efficiency and complexity), comparing them with a synthesizing quality indicator used by primary care teams in Catalonia. The *EIs* and *RI* permit a comparison of care procedures between different PHCs and health care professionals.

Defined clinical effectiveness indicators are useful to compare the morbidity of a patient population and the use of resources. Analysis of the *RI* allowed us to compare population morbidity between centres. We observed that some centres perform better on the effectiveness indicator (*SI*) despite having a higher average complexity of patient cases (*RI*). In the same manner, their costs are less than other centres with similar morbidity, which indicates that they make more efficient use of resources. The *SI* indicator in these centres had high values, indicating good quality of care based on the defined indicators. In this sense, if these results can be confirmed in subsequent analysis, the ACG® system not only allows appropriate resource allocation but also seems to be useful in professional practice, contrary to the criticism received that it could not assess how the work is being performed [20].

In the analysis of correlations between BCTs for various indicators, the *visits EI* and *costs EI* had good correlation but there was poor correlation between the *episodes EI* and *visits EI*. Although we have not found reports in the literature that simultaneously calculate these 3 indices of efficiency (episodes, visits and costs), the differences we observed in the correlations between them could perhaps be explained because the costs associated with the ACG® were calculated on the basis of the unit cost of the visit and not of the episode. This would affect the results because an episode might involve multiple visits.

Continuing our analysis of correlation between the different BCTs, the *RI* and *episodes EI* were more highly correlated than the *visits EI* or *costs EI*. As would be expected, the *RI* was more correlated with episodes, which constitute the morbidity load, and less with visits and costs. This means that as multi-morbidity increases, the health problems vary more widely from the average and therefore there is no homogeneity with respect to the development of the episodes of certain specific diseases. Further studies are required to better explain and interpret these correlations and variations between PHCs and BCTs, including more information about type of PHC (rural/urban) and the professional characteristics of general practices [15-17].

One of the goals of our study was to analyse an effectiveness indicator, the *SI*. We observed a weak correlation between the *SI* and the *visits EI*, indicating that centres with good quality indicators do not necessarily achieve them by increasing the number of visits.

There is a negative correlation between *cost EI* and *SI*. However, it should be noted that we are comparing the observed:expected cost ratio, rather than a direct cost comparison between centres. Even so, these indicators could be related to a predictor of clinical effectiveness: increased cost represents decreased effectiveness in resource usage.

The last group of correlations analysed was between the *RI* and the *SI*. The results show a trend towards centres with greater complexity and morbidity providing high-quality patient care. These results also allow us to identify and understand the resource utilization of some PHCs and BCTs and help to provide better information about the appropriateness of patient care provided. Analysis of each indicator by PHC and BCT showed that not all physicians within the same PHC act in the same way, and that the differences are related more to the physician than to the PHC. Nonetheless, it is well known that physician behaviour depends to a great extent on the complexity and morbidity of the patient population, which affects variations in the pattern of health care services provided [17,21-24].

The main limitation of our study is the data source used. The EHR may have missing information or an under-diagnosis bias; however, all PHCs use the same software that requires entering a diagnosis at each visit. Despite the potential limitations, the prevalence of morbidity as estimated by EHR is substantially higher than that reported by general population surveys, and similar to that found in population-based longitudinal studies [25]. In this sense, the use of indicators based on EHR requires that methodologies for defining patient characteristics be properly standardized, as well as those for recording the number and values of study variables, if the system is to be used to compare the efficiency and effectiveness of different PHC centres. Another potential limitation of the study was the assessment of effectiveness using the SI, an index constructed by the authors for the purpose of this study that has not been tested in other contexts; sensitivity and specificity of this index were not assessed. However, we provide detailed criteria for obtaining the SI values from standard EHR data recorded in all primary care centres in Spain [19]. Specifically, the SI index was developed using a set of indicators based on Clinical Practice Guidelines that were validated by an expert panel.

Future studies are needed to replicate the data analysis using other indicators of effectiveness that form part of international clinical practice guidelines.

One of the strengths of the present study is the use of the ACG® system developed for use in the United States and used in Spain by numerous studies [6,7,26-30]. Specifically, the ACG case-mix system was validated in Spain by a 2005 retrospective, multi-centre study of 81,873 patients [30] and in a Swedish community-based study [31]. The ACG index (*RI*) used as an indicator of morbidity burden was selected for two reasons: First, it is well established, internationally validated, and well documented for use in risk calculation [11,14]. Second, although many indicators of morbidity burden are available (e.g., Aggregated Diagnosis Groups [ADG], Major ADG, ACG-related relative weight), the *RI* would also establish the disease burden for each BCT.

The absence of significant *RI* differences between PHCs for selected diseases, confirmed by our analysis of the distributions of the prevalence of the chronic diseases included in the effectiveness indicators, allowed us to make the efficiency and effectiveness comparisons that were the primary aim of the study. Briefly, we sought to determine whether the BCTs were responding to a similar morbidity burden (RI) with any differences in efficiency (cost EI, visits EI, episodes EI) or in the effectiveness indicators (SI) routinely collected by the Catalan Health Service. The ACG® were designed to measure health status and health care resources consumed by specific groups of individuals [9,10]. Therefore, future population-based studies could be used to adjust risks of capitation and clinical management of the centres.

This study defined various indicators of efficiency, complexity and effectiveness that allowed the comparison of the behaviours of numerous PHCs and BCTs. The results of this correlation analysis are complex to interpret but permitted a comparison of PHCs effectiveness. Even so, the indicators themselves are simple and easily interpreted with the ACG® application. The use of these efficiency, effectiveness and complexity indicators should be explored in the context of assigning budget resources to each PHC, based on the morbidity of their population, to ensure that they have the needed care and quality measures in place to meet the needs of each patient. In these times of very tight budgets, budget adjustments cannot be made without considering the morbidity profile of the population attended by each PHC.

## Conclusions

Indicators of efficiency, effectiveness and complexity using ACG® permit a comparison of the care provided by different primary care centres and health providers. The relationship observed between global efficiency indicators related to episodes and costs permitted comparison of effectiveness between different PHCs. The complexity indicator (*RI*) and the *episodes EI* were also adequately correlated. The synthetic “effectiveness” indicator (*SI*) is weakly correlated with the *RI*. The use of indicators based on patient classification systems requires further study if they are to be used for purposes of comparing activities between different PHCs and health providers.

## Abbreviations

ACG®: Adjusted clinical groups; PHCs: Primary health care centres; GP: General practice physician; BCT: Basic care team; EI: Efficiency index; RI: Risk index; SI: Synthetic index; DRG: Diagnosis related groups; PHC: Primary health care centre; EHR: Electronic health record; IDIAP: Institut Universitari d’Investigació en Atenció Primària; ICPC: International classification of primary care.

## Competing interests

Research project financed by Fondo de Investigaciones Sanitarias de la Seguridad Social (Instituto Carlos III, Majahonda (Madrid), Spain; Reference: PI 08/1567).

The authors declare that they have no competing interests.

## Authors’ contributions

AS, SV, RN, CV, OP, AP, AA, BB and AR drew up the study protocol and structured the bibliographical search. AS, CV, BB and OP carried out data collection. CV, OP, QF and AS conducted the analysis and interpretation of the initial results. All authors contributed ideas, interpreted the findings and reviewed rough drafts of the manuscript. All authors approved the final versions of all manuscripts. AS is the head of the Catalan study.

## Pre-publication history

The pre-publication history for this paper can be accessed here:

http://www.biomedcentral.com/1472-6963/13/421/prepub

## Supplementary Material

Additional file 1 Components of the general synthetic index (SI): definition of each indicator of process and results. Click here for file

Additional file 2 Construction of the Synthetic Index. Click here for file
