# Supplementary material for: Relationship between efficiency and clinical effectiveness indicators in an adjusted model of resource consumption: a cross-sectional study
Source: BMC Health Serv Res. 2013 Oct 18;13:421. doi: 10.1186/1472-6963-13-421 (PMC3853183; doi:10.1186/1472-6963-13-421)
Supplement: Additional file 1 — Components of the general synthetic index (SI): definition of each indicator of process and results. [file 1472-6963-13-421-S1.docx]

**Additional file 1. Components of the general synthetic index (SI): definition of each indicator of process and results**

| 1. **Acceptable blood pressure control**   Percentage of population aged 15 years and older assigned and attended (A/A, see Concepts, #1, below) with a diagnosis of high blood pressure (BP), and with acceptable BP control.  Formula-- Numerator: Population 15 years and older A/A high BP diagnosis who have acceptable BP control.  Denominator: Total A/A population 15 years and older with high BP  Acceptable BP control: <140/90 mm Hg (in patients with diabetes <130/80 mm Hg). |
| --- |
| 1. **Acceptable diabetes control**   Percentage of A/A population aged 15 years and older with a diagnosis of arterial diabetes and have metabolic control.  Formula-- Numerator: A/A population 15 years and older with good control of diabetes. Denominator: Total A/A diabetic population 15 years and older  Diabetes control criteria: Glycated haemoglobin (HbA1c) <7% |
| **3. Blood pressure screening**  Percentage of A/A population aged 15 years and older who have at least one BP measurement in electronic health records in the past 2 years.  Formula-- Numerator: A/A population 15 years and older with at least one high blood pressure measurement. Denominator: A/A population 15 years and older without hypertension diagnosis. |
| **4. Diabetes mellitus screening**  Percentage of A/A population aged 15 years and older with at least one glycaemia recorded in electronic health records in the past 2 years.  Formula-- Numerator: A/A population 15 years and older with at least one glycaemia recorded in electronic health records. Denominator: A/A population 15 years and older without diabetes diagnosis. |
| **5. Cardiovascular risk calculation in patients aged 35-74 years with total cholesterol >200 mg/dl**  Percentage of A/A population aged 35 to 74 years with a total cholesterol value >200 mg/dl who have a cardiovascular risk assessment recorded, according to Framingham-REGICOR cardiovascular risk tables (the most recent value of the past 2 years).  Formula-- Numerator: A/A population from 35 to 74 years of age with a cardiovascular risk factor of cholesterolemia (>200 mg/dl). Denominator: Total A/A population from 35 to 74 years of age, with cholesterolemia (>200 mg/dl). |
| **6. Alcohol consumption screening (adult population)**  Percentage of A/A population aged 15 years and older with an updated record of alcohol consumption in primary care health records (past 2 years)  Formula-- Numerator: A/A population aged 15 years and older with a record of alcohol consumption. Denominator: Total A/A population aged 15 years and older.  Smoker: Person who uses tobacco or a tobacco derivative at least once a day (or weekly equivalent). |
| **7. Ex-smokers, at one year**  Percentage of A/A population aged 15 years and older with a former smoking habit, who have quit smoking (with the past 12 months)  Formula-- Numerator: A/A population aged 15 years and older who are ex-smokers at one year. Denominator: Total A/A population aged 15 years and older who are smokers and ex-smokers.  Smoker:: Person who uses tobacco or a tobacco derivative at least once a day (or weekly equivalent). Ex-smoker: Former smoker who has abstained from smoking for a year or more. |
| **8. Ischemic heart disease patients with adequate antiplatelet treatment**  Percentage of A/A population aged 15 years and older who have ischemic heart disease and are receiving appropriate antiplatelet treatment.  Formula-- Numerator: A/A population aged 15 years and older with ischemic heart disease diagnosis and who take antiplatelet treatment. Denominator: Total A/A population aged 15 years and older with a diagnosis of ischemic heart disease. |
| **9.. Ischemic heart disease patients with low density lipoprotein <100 mg/ml**  Percentage of A/A population aged 15 years and older who have ischemic heart disease and appropriate control of dyslipidaemia.  Formula-- Numerator: A/A population aged 15 years and older with ischemic heart disease diagnosis and a low density lipoprotein level <100 mg/ml.  Denominator: Total A/A population aged 15 years and older with ischemic heart disease diagnosis. |
| **10. Cardiac arrhythmia patients with atrial fibrillation and anticoagulation treatment**  Percentage of A/A population aged 15 years and older who have atrial fibrillation and take anticoagulant medication  Formula-- Numerator: A/A population aged 15 years and older with atrial fibrillation diagnosis who take anticoagulant medication  Denominator: Total A/A population aged 15 years and older with atrial fibrillation diagnosis |
| **11..Population >74 years, including home health care**  Percentage of A/A population aged 74 years and older attended by Homecare Program  Formula-- Numerator: number of assigned patients aged 74 or older attended by the Homecare Program. Denominator: Total number of A/A patients aged 74 or older. |
| **12..Population >74 years, including home health care, with evaluation**  Percentage of A/A population aged 74 years and older attended by Homecare Program with a geriatric evaluation.  Formula-- Numerator: number of A/A patients aged 74 or older attended by the Homecare Program with an evaluation. Denominator: Total number of A/A patients aged 74 or older attended by the Homecare Program. |
| **13..Flu vaccine administered in the assigned population aged 60 years or older**  Percentage of A/A population aged 60 years and older correctly vaccinated against influenza.  Formula-- Numerator: A/A population aged 60 years and older attended and correctly vaccinated against influenza. Denominator: Total A/A population aged 60 years and older. |
| **14..Patients with chronic obstructive pulmonary disease and received pneumococcal vaccine**  Percentage of A/A population aged 60 years and older correctly vaccinated with pneumococcal vaccine.  Formula-- Numerator: A/A population aged 60 years and older vaccinated with pneumococcal vaccine. Denominator: Total A/A population aged 60 years and older. |
| **15..Use of generic pharmaceutical drugs**  Percentage of generic drugs prescribed (in the past year) compared to all drugs prescribed.  Formula-- Numerator: Number of drugs prescribed by generic name. Denominator: Number of drugs of prescribed. Multiplied by 100.  Generic drug: a drug product that is comparable to brand/reference listed drug product in dosage, form, strength, route of administration, quality and performance characteristics, and intended use. |
| **16..Use of new medications with limited added value**  Percentage of new drugs prescribed with a limited added value (marketed in the past year).  Formula-- Numerator: Number of new drugs prescribed with a limited added value. Denominator: Total number of drugs prescribed.  Added value: The Catalan Health Service establishes the category of “new drug without adding value” according to available evidence. |
| **17. Average cost per defined daily dose of proton pump inhibitors**  Average cost per defined daily dose (DDD) of proton pump inhibitor prescribed.  Formula-- Numerator: Cost of DDDs of proton pump inhibitors prescribed. Denominator: Total number of DDDs for proton pump inhibitors drugs prescribed. |
| **18..Average cost per defined daily dose of statins**  Average cost per DDD of statins  Formula-- Numerator: Cost of DDD statins prescribed. Denominator: Total number of DDDs of statins prescribed. |
| **19. Average cost per defined daily dose of angiotensin-converting enzyme inhibitors (ACEI) and angiotensin II receptor antagonist (Angio II)**  Average cost per DDD of ACEI and Angio II  Formula-- Numerator: Cost of DDDs of ACEI prescribed. Denominator: Number of DDDs of Angio II + ACEI. |
| **20. Average cost per defined daily dose of serotonin re-uptake inhibitors and new-generation antidepressants**  Average cost per DDD of recommended serotonin re-uptake inhibitors (SRI) and new-generation antidepressant drugs prescribed.  Formula-- Numerator: Cost of DDDs of SRI and new-generation antidepressants prescribed. Denominator: Number of DDDs of SRI and new-generation antidepressants prescribed. |

**Concepts:**

1. Assigned and attended (A/A): individuals who are assigned to a primary care team in general practice, in accordance with the current allocation criteria in Catalonia and have had one or more contacts with the primary health care centre within the defined study period.

2. Defined daily dose: (DDD): the assumed average maintenance dose per day, according to the WHO assigned value, for a drug used for its main indication in adults. For instance, the DDD for atorvastatin is 10 mg.

3. Number of DDDs: drug utilization expressed in DDD, calculated by converting the number of units reported in the data into the number of DDDs, according to the DDD methodology. For instance, 100 DDDs of atorvastatin (DDD = 10) corresponds to 1,000 mg.

**References:**

1.Technical report. Purchase of services 2007. **Contracts for primary health care teams** (available in Catalan). Barcelona: Catalan Health Department; 2007.

2.Iglesias-Pérez B, Cortés-Pérez P, Fina-Avilés F, Mendez-Boo L, Ferran- Mercadé M, Medina- Peralta M, Serrano- Carro B, Casajuana Brunet J, Calero Muñoz S, Morros Pedrós R, AmatCamats G; **Synthetic indicator of Quality of care of primary care teams 2008**. Barcelona: Catalan Health Institute, 2008.

Available in Catalan from: <http://www.gencat.net/ics/professionals/pdf/EQA_2008.pdf>

3. WHO Collaborating Centre for Drug Statistics Methodology. Available at: <http://www.whocc.no/>
