# Supplementary material for: Relationship between efficiency and clinical effectiveness indicators in an adjusted model of resource consumption: a cross-sectional study
Source: BMC Health Serv Res. 2013 Oct 18;13:421. doi: 10.1186/1472-6963-13-421 (PMC3853183; doi:10.1186/1472-6963-13-421)
Supplement: Additional file 2 — Construction of the Synthetic Index. [file 1472-6963-13-421-S2.docx]

**Additional file 2. Construction of the Synthetic Index**

Indicators 1 to 14 were obtained from a universal electronic health records database (*Oficina médica informatizada en Atención primaria*, OMI-ap®, Stacks, <http://www.stacks.es>) and indicators 15 to 20 from the Pharmacy Prescription Invoices received by the Catalan Health Department.

Synthetic Index values were calculated for the indicators:

A) Average value for each general practitioner for indicators 1 to 15.

B) Average value for indicators 16 to 20 (value B). These were calculated inversely to achieve the highest possible value (100-B).

The weighted average of A (with weight 15) and 100-B (weight 5) was calculated as follows:

Formula:

$$\begin{matrix} A=\frac{I1+I2+...+I15}{15} \\ B=\frac{I16+I17+...+I20}{5} \\ SI=\frac{15A+5(100-B)}{20} \end{matrix}$$

*I:* indicator

*SI:* synthetic index

Simplified as

$$SI=\frac{I1+I2+...+I15+(100-I16)+...+(100-I20)}{\begin{matrix} 20 \\ \end{matrix}}$$

For the following indicators (detailed in Appendix 1):

| No. | INDICATOR LABEL |
| --- | --- |
| I1 | Controlled blood pressure |
| I2 | Controlled diabetes |
| I3 | Hypertension screening |
| I4 | Diabetes mellitus screening |
| I5 | Cardiovascular risk calculation coverage, adult population (35-74y) with cholesterolemia (Total >200 mg/dl) |
| I6 | Alcohol screening, adult population (35-74y) |
| I7 | Ex-smokers at 1 year |
| I8 | Patients with ischemic heart disease and appropriate antiplatelet treatment |
| I9 | Patients with ischemic heart disease and LDL<100 mg/ml |
| I10 | Patients with ACxFA and anticoagulants treatment |
| I11 | Total population >74 years old, included in home health care programme |
| I12 | Total population >74 years old, included in home health care programme, with evaluation |
| I13 | Flu vaccine coverage in over-60 population |
| I14 | COPD + pneumococcal vaccine coverage |
| I15 | Use of generic drugs |
| I16 | Use of new drugs with limited added value |
| I17 | Average cost per DDD proton pump inhibitors |
| I18 | Average cost per DDD: statins |
| I19 | Average cost per DDD: ACEI + ARA II |
| I20 | Average cost per DDD: SRI + new-generation antidepressants |
